# Cellular and Molecular Mechanisms of Kidney Development: From the Embryo to the Kidney Organoid

**DOI:** 10.3389/fcell.2020.00183

**Published:** 2020-03-24

**Authors:** Niloofar Khoshdel Rad, Nasser Aghdami, Reza Moghadasali

**Affiliations:** ^1^Department of Stem Cells and Developmental Biology, Cell Science Research Center, Royan Institute for Stem Cell Biology and Technology, ACECR, Tehran, Iran; ^2^Department of Developmental Biology, University of Science and Culture, Tehran, Iran; ^3^Department of Regenerative Medicine, Cell Science Research Center, Royan Institute for Stem Cell Biology and Technology, ACECR, Tehran, Iran

**Keywords:** differentiation, kidney development, organoid, renal progenitors, signaling pathways

## Abstract

Development of the metanephric kidney is strongly dependent on complex signaling pathways and cell–cell communication between at least four major progenitor cell populations (ureteric bud, nephron, stromal, and endothelial progenitors) in the nephrogenic zone. In recent years, the improvement of human-PSC-derived kidney organoids has opened new avenues of research on kidney development, physiology, and diseases. Moreover, the kidney organoids provide a three-dimensional (3D) *in vitro* model for the study of cell-cell and cell-matrix interactions in the developing kidney. *In vitro* re-creation of a higher-order and vascularized kidney with all of its complexity is a challenging issue; however, some progress has been made in the past decade. This review focuses on major signaling pathways and transcription factors that have been identified which coordinate cell fate determination required for kidney development. We discuss how an extensive knowledge of these complex biological mechanisms translated into the dish, thus allowed the establishment of 3D human-PSC-derived kidney organoids.

## Introduction

The mammalian kidney is one of the most complex organs in the body. The kidney is the major homeostatic organ necessary for pH and electrolyte regulation, and maintenance of overall fluid balance. In addition to these excretory functions, the kidney produces several hormones and humoral factors such as renin, erythropoietin, calcitriol (1,25-dihydroxycholecalciferol) and prostaglandins (Santoro et al., 2015). Kidney function depends on nephrons, the structural and filtration unit of the kidney, that are composed of more than 20 different specialized cells (Al-Awqati and Oliver, 2002). In the human kidney, nephrons are generated only during nephrogenesis and *de novo* nephron formation continues until 36 weeks of gestation (Romagnani et al., 2013; Ahmadi et al., 2019a). *In vitro* re-creation of these complex structural units of the kidney is a challenging issue; however, there has been some success in the past decade. A defined culture system drives the differentiation of human pluripotent stem cells (hPSCs) into kidney organoids by recapitulating the developmental processes. Generation of human PSCs-derived kidney organoids depends on cell–cell communication between multiple distinct progenitor populations that lie adjacent to each other (Morizane et al., 2015; Garreta et al., 2019; Homan et al., 2019). This review focuses on major signaling pathways and transcription factors that coordinate cell fate determination of renal progenitor cells. We intend to discuss the ways in which cell communications between nephron progenitor cells (NPCs), ureteric bud progenitor cells (UBPCs), endothelial and stromal cells during organogenesis lead to a fully patterned and vascularized kidney tissue, and how a deep knowledge of these biological mechanisms translated into the dish, thus allowed the establishment of PSCs-derived kidney organoids.

## Spatial Organization and Early Patterning of the Kidney-Forming Mesoderm

**FIGURE 1:**
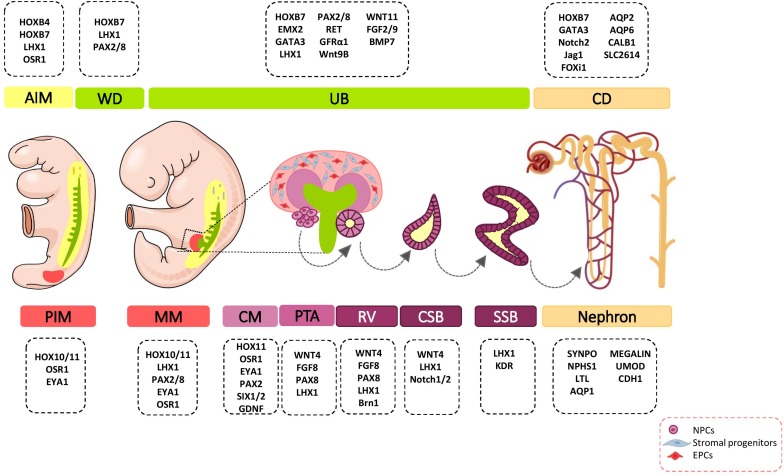
Major genetic markers involved in kidney development. The cell fate decision of renal cells are coordinately controlled with different genetic markers during nephrogenesis. AIM, anterior intermediate mesoderm; AQP, aquaporin; BMP, bone morphogenetic protein; Brn1, Bruno-like1; CALB1, calbindin; CD, Collecting duct; CM, cap mesenchyme; CSB, comma-shaped body; EMX, empty spiracles homolog; Eya1, eyes absent 1; FGF, fibroblast growth factor; FOXi1, forkhead box protein i1; FRS2α, fibroblast growth factor receptor substrate 2α; GATA, *trans*-acting T-cell-specific transcription factor; GDNF, glial cell–derived neurotrophic factor; GFRα1, glial cell line derived neurotrophic factor family receptor α1; Jag1, Jagged 1; HOX, homeobox; IM, intermediate mesoderm; Kdr, kinase insert domain protein receptor; LHX1, LIM-class homeodomain 1; LTL, lotus tetragonolobus lectin; MM, metanephric mesenchyme; NPCs, nephron progenitor cells; NPHS1, nephrosis 1; Osr1, odd skipped related 1; PAX, paired box protein; PI3K, phosphoinositide 3-kinases; PIM, posterior intermediate mesoderm; PTA, pre-tubular aggregate; RV, renal vesicle; SIX2, sine oculis-related homeobox 2; SLC2614, solute carrier 2614; SSB, S-shaped body; SYNPO, synaptopodin; TGFβ, transforming growth factor beta; UB, ureteric bud; UMOD, uromodulin; WNT, wingless-type mouse mammary tumor virus integration site; WD, Wolffian duct.

During organogenesis, the intermediate mesoderm (IM) gives rise to three types of excretory organs: pronephros, mesonephros, and metanephros. The metanephric kidney remains for the period after birth and forms the definitive mature organ. Metanephros differentiates as the result of interaction between the metanephric mesenchyme (MM), which is derived from the most posterior intermediate mesoderm (PIM), and the ureteric bud (UB) lineage that includes the collecting system that is derived from a more anterior IM (Taguchi et al., 2014; Takasato and Little, 2015). PIM have a multi-potent precursor population that give rise to nephron segments and interstitial stromal cells. The signals that specify the early kidney field along the body axes have received more attention. Several transcriptional regulators such as homeobox (Hox) paralogs, LIM1 (LIM-class homeodomain1), odd skipped related 1 (OSR1), PAX2/8 (Paired box protein 2/8), and eyes absent 1 (EYA1) have been shown to play major roles in early patterning and specification of the developing kidney (Figure 1) (Bouchard et al., 2002). These events lead to the formation of multiple distinct renal progenitor populations within the nephrogenic niche.

### The Homeobox (*Hox*) Genes

The *Hox* genes have an important role in anterior-posterior patterning of the body. From these, 28 of the 39 *Hox* genes are expressed in the developing kidney (Patterson and Potter, 2004). Given that the Hox proteins have intrinsically weak DNA-binding affinity, their interaction with cofactors is critical for target selectivity (Gong et al., 2007). Thus, interaction of *Hox* genes with regulatory partners such as Pax2, Eya1, and SMADs [*Caenorhabditis elegans* SMA (“small” worm phenotype) and *Drosophila* MAD (“Mothers Against Decapentaplegic”)] is necessary for kidney mesoderm specification (Gong et al., 2007; Preger-Ben Noon et al., 2009). *Hoxb4* plays key roles in the establishment of the kidney morphogenetic field anterior border (Preger-Ben Noon et al., 2009) and nephric duct specification (Attia et al., 2012). Retinoic acid (RA) signaling in the anterior IM stimulate the expression of *Hoxb4*. *Hoxb4* confers competence on IM cells to respond to inductive signals from neighboring tissues. Cooperation of Hoxb4 with SMADs induces expressions of Lim1 and Pax2 in IM cells (Preger-Ben Noon et al., 2009). Another *Hox* gene, *HoxB7*, is expressed from the early stages in the nephric duct to terminal differentiation of UB derivatives, including the ureter, pelvis, calyces, and collecting ducts (Argao et al., 1995; Srinivas et al., 1999). However, the direct downstream targets of Hoxb7 in these cells is unknown. Hoxa11 and Hoxd11 expressions are restricted to the PIM, which develops into MM. Hoxd11 is expressed in both cap mesenchyme (CM) and cortical stroma (Mugford et al., 2008a), and activates several metanephric specific markers, including sine oculis-related homeobox 2 (SIX2) (Mugford et al., 2008a), glial cell-derived neurotrophic factor (GDNF), forkhead/winged helix transcription factor (FOXD1) (Patterson et al., 2001), and pre-B-cell leukemia transcription factor 1 (PBX1) (Moens and Selleri, 2006). Hox11 function is required for generation of NPCs, stromal progenitor cells, and induction of UB branching morphogenesis. Studies have shown that Hox11 paralogs interact with Pax2 and Eya1 to induce transcription of direct downstream targets such as SIX2 and GDNF (Wellik et al., 2002; Gong et al., 2007). Therefore, the spatiotemporal pattern of Hox11 expression indicates that it has a key role in MM patterning. The results of a study have shown that although Hox10 and Hox11 expression patterns mostly overlap, Hox10 displays additional expression in the FOXD1-expressing cortical stromal cells. Hox10 has an essential role in appropriate integration and further differentiation of stromal progenitor cells in the developing metanephric kidney (Yallowitz et al., 2011).

### LIM-Class Homeodomain 1 (Lim1)

LIM-class homeodomain 1 (Lim1) is a transcription factor encoded by the *LHX1* gene in humans. Lim1 is an early marker for kidney organogenesis. This gene is a direct downstream target for the RA signaling pathway to IM specification and patterning (Osafune et al., 2002; Cartry et al., 2006; Wingert et al., 2007). During renal development, Lim1 is expressed in different stages - the IM; nephric duct; pro- and mesonephros; UB; pre-tubular aggregates (PTA); comma- and S-shaped bodies; and podocytes. Its expression pattern suggests that Lim1 has distinct functions in several steps of kidney organogenesis. To this end, Lim1 affects expression of several key genes and regulates cell fate specification. According to research, Lim1 regulates its own expression and the expressions of Pax2, *E*-cadherin, WNT9b, and Ret in the nephric duct, thereby influencing early specification of the IM, nephric duct elongation, and UB outgrowth (Tsang et al., 2000; Kobayashi et al., 2005; Pedersen et al., 2005). This cell fate-specifying transcription factor regulates the patterning of renal vesicles (RV) by transcriptional activation of Brn1 (Bruno-like1) and EphA4 (Chen et al., 2006).

### Odd Skipped Related 1 (Odd1 or Osr1)

OSR1 is a zinc-finger DNA-binding protein that is broadly expressed in the IM and MM (James, 2006). OSR1 is one of the earliest genetic markers that is expressed in the MM and UB lineages. OSR1 is specifically required for establishment of the MM. Early OSR1 expressing cells are a multi-potent precursor population that give rise to nephron and interstitial mesenchyme progenitors (Mugford et al., 2008b). In the nephrogenic lineage, OSR1 expression is downregulated from the RV stage (Xu et al., 2014). OSR1 regulates the expressions of several key genes (*LHX1*, *PAX2*, *EYA1*, *SIX2*, *GDNF*, Cbp/p300-interacting transactivator 1 [*CITED1*], and Spalt like transcription factor 1 [*SALL1*]) in the nephrogenic mesenchyme (James, 2006; Xu et al., 2014, 2016). OSR1 interacts synergistically with other factors such as Wilm’s tumor (WT1) and SIX2 to regulate MM specification and NPC pool maintenance (Xu et al., 2014, 2016). OSR1-dependent transcriptional activation of LHX1 might regulate expression of foot process and podocyte junction-associated genes that result in podocyte differentiation (Tomar et al., 2014).

### Paired Box Proteins (*PAX2/8*)

The paired box proteins (PAX2/8) transcription factors are earlier genetic markers expressed in the IM. RA signaling and low levels of bone morphogenetic protein (BMP) signaling from neighboring tissues induce the expressions of *PAX2/8* genes in the kidney-forming mesoderm (James and Schultheiss, 2005; Cartry et al., 2006; Fleming et al., 2013). PAX2 transcripts and proteins are found in multiple stages of the developing kidney, including the IM, nephric duct, UB, MM, and CM. Subsequently, its expression in MM derivatives is downregulated (Ryan et al., 1995; Mugford et al., 2008a) and becomes restricted to UB derivatives (Cai et al., 2005). In the early stage of kidney development, PAX8 and PAX2 are co-expressed. As development proceeds, the PAX8 mRNA and protein disappear, and are expressed again in the RV stage (Narlis et al., 2007). PAX2/8 can affect signaling in the developing kidney by transcriptional regulation of *GATA3* (Trans-acting T-cell-specific transcription factor), *LIM1* (Narlis et al., 2007; Boualia et al., 2013), *RET* (Bouchard et al., 2002), *SALL1* (Ranghini and Dressler, 2015), *SIX2*, *GDNF* (Brophy et al., 2001), *WNT4* (Torban et al., 2006), and secreted frizzled-related protein 2 (*SFRP2*) (Brophy et al., 2003) genes during multiple steps. The results of studies indicate that both *PAX2/8* are critical for cell survival, branching morphogenesis, and nephron specification (Bouchard et al., 2002; Narlis et al., 2007).

### Eyes Absent Homolog 1 (EYA1)

EYA1 is a transcription regulator with threonine phosphatase activity. EYA1 is expressed in the PIM, MM, and CM. As nephrogenesis proceeds, its expression is gradually decreased (Xu et al., 2015). EYA1 forms a transcriptional complex with homeodomain genes during multiple stages of nephrogenesis. Eya1-Six1-Dach and Eya1-Hox11-Pax2 complexes during the early stages of MM activate expressions of SIX2 and GDNF in the mesenchymal progenitors (Li et al., 2003; Gong et al., 2007; Xu et al., 2015). Thereafter, the SIX2-Eya1-Myc complex is critical for expansion of the multi-potent nephron progenitor pool (Xu et al., 2015).

Early patterning of kidney-forming mesoderm leads to the formation of multiple distinct renal progenitor populations within the nephrogenic niche. Kidney organogenesis depends on cell–cell communication between these populations that lie adjacent to each other.

## Renal Progenitor Cells

Pioneering studies revealed that the renal nephrogenic niche includes at least four major self-renewing, multi-potent progenitor cell populations: UBPCs, NPCs, stromal progenitors, and endothelial progenitor cells (EPCs; Figure 1). Kidney organogenesis, like the organogenesis of all other organs, is dependent on the migration of external cells from different embryonic tissues into the developing kidney (Bronner-Fraser and Fraser, 1988; Schmidt-Ott et al., 2006; Guillaume et al., 2009). Spatiotemporal multicellular interactions and precise orchestration of signals between several distinct cell populations have important roles in the successful induction, maintenance, and differentiation of all cell types of the kidney.

### Nephron Progenitor Cells (NPCs)

**FIGURE 2:**
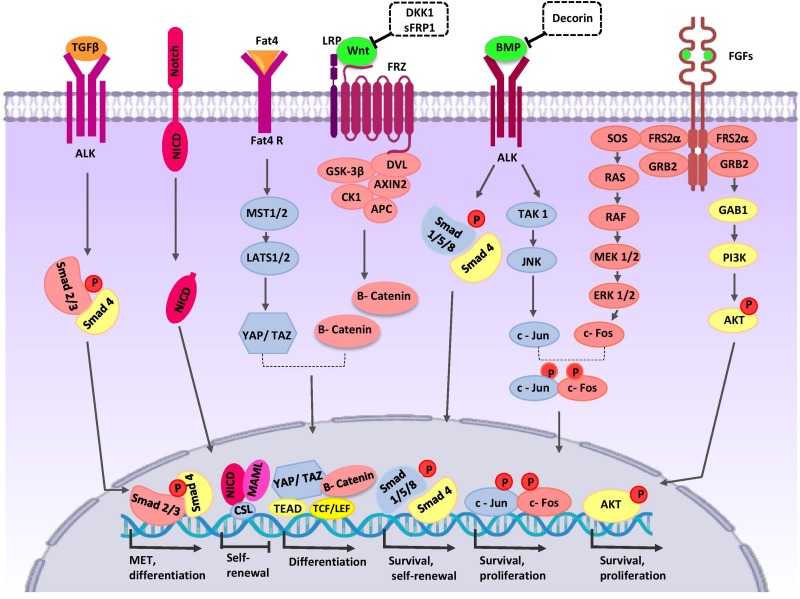
Crosstalk between major signaling pathways during nephron progenitor cell (NPC) differentiation. WNT9b, BMP7, and FGF2/9 are secreted by UB cells (green color). Stromal cells secret Decorin, SFRP1, Fat4, and TGFβ2 (orange color). High levels of WNT9b/β-catenin increase expression of the differentiation-specific genes (*PAX8, C1qdc2*, and *WNT4*) of nephron progenitor cells (NPCs). Fat4/Hippo signaling amplifies β-catenin activity. On the other hand, WNT signaling can be inhibited by SFRP1 and DKK1 to arrive appropriate number of nephrons in a definitive kidney. The BMP7/SMAD1/5 signaling pathway promotes differentiation of NPCs. Decorin antagonizes BMP7/SMAD signaling in NPCs. BMP7 activates proliferation by the TAK1-JNK-JUN cascade. FOS activation is regulated by FGF9. AP1 (a dimeric transcription factor composed of Jun and FOS) acts as a point of collaboration between the BMP7 and FGF9 signaling pathways. AP-1 activates transcription of a variety of genes (*MYC, BCL-2*, and *p53*) related to the cell cycle and anti-apoptotic events; thereby, it regulates survival and proliferation of NPCs. The FGF/RAS-MAPK, FGF/PI3K/AKT signaling pathway promotes survival and proliferation of NPCs. After binding of Notch2 to Notch ligands, NICD is released into the cytoplasm and translocates to the nucleus where the complex decreases self-renewal specific gene expression and primes NPCs for differentiation. TGFβ2 is required for MET-related gene expression during NPCs differentiation. AKT, protein kinase B; ALK, anaplastic lymphoma kinase; AP1, activator protein 1; APC, adenomatous polyposis coli; BMP, bone morphogenetic protein; CK1, casein kinase 1; Csl, CBF1/RBP-J, Su(H), Lag-1, the mammalian, fly, and worm orthologous proteins; DKK1, DKK1, Dickkopf-1; DVL, homologous to drosophila Dishevelled; EC, endothelial cells; ERK, extracellular signal-regulated kinase; Fat4, tumor suppressor homolog 4; FGF, fibroblast growth factor; Frz, Frizzled; GAB1, Grb2-associated binder 1; GSK-3β, glycogen synthase kinase 3β; GRB2, growth factor receptor-bound protein 2; JAK, Janus kinase; JNK, c-Jun N-terminal kinase; LATS1, large tumor suppressor homolog 1; LEF, lymphoid enhancing factor; LRP, low-density lipoprotein receptor-related protein; MAML, mastermind-like; MAPK, mitogen activated protein kinase; MEK, mitogen activated protein kinase; MET, mesenchymal to epithelial transition; MST1/2, Mammalian sterile 20-like kinases; NICD, notch intracellular domain; P, phosphate group; PI3K, phosphatidylinositol 3-kinase; RAS/RAF, Rat sarcoma/rapidly accelerated fibrosarcoma; SFRP1, secreted frizzled-related protein; Smad, Caenorhabditis elegans SMA (“small” worm phenotype) and Drosophila MAD (“Mothers Against Decapentaplegic”); SOS, Son of Sevenless; TAK1, TGF β-activated kinase; TCF, T-cell factor; TEAD, transcription factor family member; TGF-β, transforming growth factor-β; WNT, wingless-type mouse mammary tumor virus integration site; YAP, yes-associated protein.

NPCs harbor the capacities of both self-renewal and differentiation to maintain the nephron progenitor pool and generation of all epithelial cells of nephrons. NPCs undergo mesenchymal-epithelial-transition (MET) and sequential morphological alterations to form the PTA that differentiate into RV, comma- and S-shaped bodies, and mature nephrons. One nephron is composed of more than 20 different cell types that include podocyte cells, the proximal tubule, loop of Henle, distal tubule, and connecting tubule cells (Al-Awqati and Oliver, 2002). The modes of proliferation and differentiation of NPCs are coordinately controlled during nephrogenesis. For this purpose, NPCs-specific transcription factors CITED1, PAX2, EYA1, SIX2, SALL1, and WT1 specify cell phenotypes (Lindström et al., 2018). NPCs population in the CM are divided into the self-renewing CITED1^+^/SIX2^+^ compartment and CITED1^–^/SIX2^+^ induced compartment that progress toward epithelialization (Brown et al., 2013). SIX2 expression is controlled by upstream signaling proteins such as the Pax2/Eya1/Hox11 complex (Gong et al., 2007). SIX2 maintains the un-differentiated cell state of NPCs, and its expression is progressively decreased in the following steps of kidney organogenesis (Park et al., 2012). Several lines of evidence indicate that SIX2 regulates its own expression and the expressions of LHX1, OSR1, WT1, GDNF, FGF8, and WNT4 in NPCs and thereby regulates cell maintenance and self-renewal (Brodbeck et al., 2004; Self et al., 2006; Park et al., 2012; Xu et al., 2014). WT1 is another transcription factor that is important in regulation of self-renewal, MET, and differentiation of NPCs (Hartwig et al., 2010; Fanni et al., 2011). WT1, by inhibition of BMP7/pSMAD signaling, can repress apoptosis in MM (Motamedi et al., 2014). WT1 directly activates growth arrest-specific 1 (GAS1) transcription and promotes NPCs proliferation via the fibroblast growth factor (FGF) stimulated phosphoinositide 3-kinase (PI3K)-Akt signaling pathway (Kann et al., 2015). FGF16/20 are direct transcriptional targets of WT1 (Motamedi et al., 2014). The three-dimensional (3D) arrangement of NPCs and communication with other cells in the developing nephrogenic zone is critical for the cell fate decisions of these cells (Figure 2).

### Ureteric Bud Progenitor Cells (UBPCs)

**FIGURE 3:**
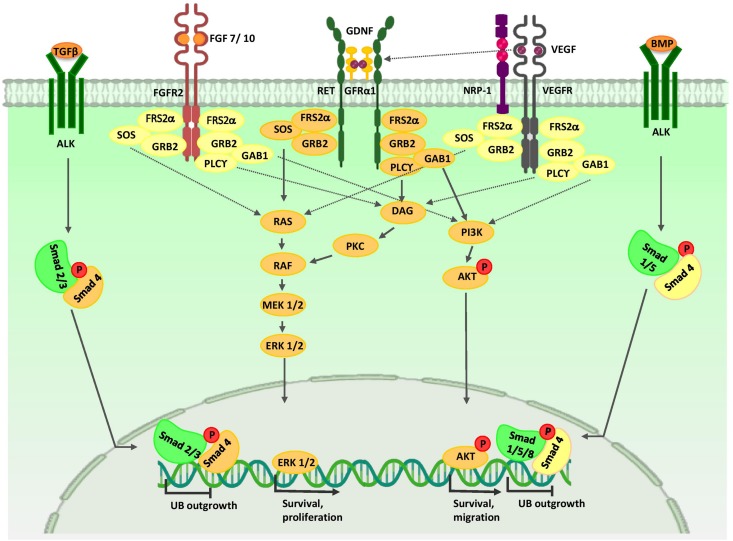
Major signaling pathways in ureteric bud morphogenesis. Branching morphogenesis is tightly regulated by different growth factors such as GDNF, VEGF, and FGFs. GDNF and VEGF are secreted from the MM and FGF7/10 is produced by stromal cells. Binding of these growth factors to their tyrosine kinase receptors activates three major signaling pathways: RAS/MAPK, DAG/PKC/MAPK, and PI3-K/AKT. Thus, they stimulate mitotic proliferation, survival, and migration of UB cells. VEGF-A induces RET activation. Members of the TGF-β super-family, including BMP4 and TGFβ, which are expressed by mesenchymal cells that surround UB, inhibit UB outgrowth in a Smad-dependent manner. AKT, protein kinase B; ALK, anaplastic lymphoma kinase; BMP, bone morphogenetic protein; ERK, extracellular signal-regulated kinase; DAG, diacylglycerol; DVL, homologous to drosophila Dishevelled; EC, endothelial cells; FGF, fibroblast growth factor; FGFR, fibroblast growth factor receptor; FRS2α, fibroblast growth factor receptor substrate 2α; GAB1, Grb2-associated binder 1; GDNF, glial cell-derived neurotrophic factor; GFRα1, glial cell line-derived neurotrophic factor family receptor α1; GRB2, growth factor receptor-bound protein 2; MEK, mitogen activated protein kinase; NRP1, neuropilin 1; P, phosphate group; PI3K, phosphatidylinositol 3-kinase; PKC, protein kinase C; PLC-γ, Phospholipase C-γ; RAS/RAF, Rat sarcoma/rapidly accelerated fibrosarcoma; Smad, Caenorhabditis elegans SMA (“small” worm phenotype) and Drosophila MAD (“Mothers Against Decapentaplegic”); SOS, Son of Sevenless; Smad, Caenorhabditis elegans SMA (“small” worm phenotype) and Drosophila MAD (“Mothers Against Decapentaplegic”); TGF-β, transforming growth factor-β; UB, ureteric bud; VEGF, vascular endothelial growth factor; VEGFR, vascular endothelial growth factor receptor.

Anterior intermediate mesoderm (AIM) commit to the UB lineage, including the collecting system (Ohmori et al., 2013). The collecting duct have critical roles in electrolyte and fluid balance, and acid-base homeostasis (Costantini and Kopan, 2010). The collecting duct consists of two highly specialized cell types, principal cells and intercalated cells. Both populations are derived from bi-potent UB precursors located at the UB tips (Al-Awqati, 2013). vHNF1 (Garcia-Villalba et al., 2009), EMX2 (Empty spiracles homolog 2) (Miyamoto et al., 1997), PAX2 (Dressler et al., 1990), LHX1 (Karavanov et al., 1998), GATA3 (Grote, 2005; Grote et al., 2008), RET (Pachnis et al., 1993), WNT11 (Majumdar, 2003), and Vsnl1 (Bridgewater et al., 2011) are expressed in the UB tips and they specify its cell fate. Results from studies indicate that a sub-population of UBPCs, which express the ΔNp63 isoform (N-terminus truncated p63) is dedicated to generating cortical intercalated cells (El-dahr et al., 2017). FOXi1 and a disintegrin and metalloproteinase domain 10 (Adam10)/Notch signaling pathway play critical roles in intercalated and principal cell fate decision in the collecting duct, respectively (Al-Awqati and Schwartz, 2004; Jeong et al., 2009; Vidarsson et al., 2009; Guo et al., 2015). In response to paracrine signals from neighboring tissues, UB precursors undergo morphological changes and coordinated cell movements to form the collecting duct (Figure 3).

### Stromal Progenitor Cells

The interstitial stroma is defined as a heterogeneous population of cells that serve both as a supportive environment and a source of dedicated cells that produce extracellular matrix (ECM) and associated signaling molecules. Stromal progenitors are spindle-shaped cells that encompass the anterior part of NPCs in the MM and later localize around nascent UBs and nephrons. The stromal cells not only provide structural support but also regulate the development of neighboring cells. Cellular origins of the FOXD1^+^ cortical stromal cell lineage arise from multipotential Osr1^+^ cells in the IM (Mugford et al., 2008b). This multipotent stromal progenitor population is characterized by expressions of FOXD1, PBX1, POD1, SALL1, retinoic acid receptors (RARs), and FAT4 (Das et al., 2013; Bagherie-lachidan et al., 2015; Ohmori et al., 2015). Some researchers have demonstrated that renal stromal cells may be derived from migrating cells of other tissues such as paraxial mesoderm and neural crest that integrate into the FOXD1^+^ compartment of the MM (Bronner-Fraser and Fraser, 1988; Guillaume et al., 2009). TBX18 expressing cells are another population of multi-potent mesenchymal progenitors in the metanephric kidney that contribute to the ureteric mesenchyme and renal interstitial cells (Bohnenpoll et al., 2013). Cells derived from mesenchymal progenitors contribute to different types of stromal cells, including interstitial fibroblasts, vascular smooth muscle cells, renin producing cells, pericytes, and mesangial cells (Kobayashi et al., 2014).

### Endothelial Progenitor Cells (EPCs)

Renal vasculature plays a significant role in the development and function of the kidney. It has been shown that endothelial cells (EC) are important not only for delivery of oxygen and micronutrients, but also for paracrine signals that are distributed to other cells in the nephrogenic niche that promote kidney organogenesis (Munro et al., 2017). Development of the renal vasculature proceeds synchronously with nephrogenesis and occurs through two main mechanisms, vasculogenesis and angiogenesis (Mukherjee et al., 2017). Sprouting angiogenesis of pre-existent vessels plays a key role in formation of the major vessels (Nishimura et al., 2016). The renal vasculature arises predominantly from formation of *de novo* vessels via differentiation of endothelial progenitors (angioblasts) (Sequeira-lopez et al., 2015). Results of recent studies suggest that there are different populations of both intra- and extra-renal EPCs (Mugford et al., 2008b; Munro et al., 2017). Previously, a population of FLK1-expressing cells (vascular endothelial growth factor [VEGF]-A receptor, VEGFR2 [FLK1, KDR]) in the periphery of the induced mesenchyme and adjacent to the stalk of the UB have been identified (Robert et al., 2018). These cells are most probably derived from OSR1^+^ multi-potential progenitors within the IM (Mugford et al., 2008b). In the following stages of development, these KDR^+^ cells undergo changes to immature intermediate melanoma cell adhesion molecule (MCAM^+^, CD146^+^) cells, and at the end of the developmental period, platelet/endothelial cell adhesion molecule-1 (PECAM^+^, CD31^+^) mature vascular cells (Homan et al., 2019). Furthermore, a population of c-Kit^+^ endothelial progenitors reside within the cortical stromal compartment. Studies indicate that this progenitor population has migrated from the aorta-gonad-mesonephros (AGM) region to the early MM. UB cells secrete stem cell factor (SCF), a c-Kit ligand and thereby promote survival, migration, and tube formation of EC (Schmidt-Ott et al., 2006; Homan et al., 2019). Another source of endothelial progenitors is a subpopulation of FOXD1^+^ renal stromal cells that are incorporated into the peritubular capillary. These cells play a critical role in the proper spatial distribution of renal vessels (Sims-lucas et al., 2013; Mukherjee et al., 2017). A subset of MCAM^+^ progenitors that are derived from a FOXD1^+^ renal stromal population are incorporated into endothelial structures (Pärssinen et al., 2016). Results of a transcriptomic study have revealed that a subpopulation of SALL1^+^/SIX1^+^ NPCs reside in the second-trimester of human fetal kidneys and co-express the CD31 mature endothelial marker (Low et al., 2019).

## Regulation of Nephron Progenitor Cell (Npc) Fate

Many studies identified biological processes and signaling pathways that regulate cell fate decisions of NPCs. We intend to discuss the critical role of the wingless-type mouse mammary tumor virus integration site (Wnt) protein family, FAT4, Hippo, BMPs, FGFs, Notch, and Hedgehog/transforming growth factor beta (TGFβ) signaling pathways and explain how cross-talk between them determines the cell fate of NPCs (Figure 2).

### Wnt Family Signaling Pathways

WNT9b/β-catenin signaling is one of the major signals that mediate nephron progenitor renewal and differentiation (Karner et al., 2011). The activity of β-catenin in NPCs is controlled by signals from the UB and cortical stroma. Low levels of β-catenin increase expression of self-renewing genes and promote expansion of the NPC pool. High levels stimulate transcription of several differentiation-specific genes such as *PAX8, C1qdc2*, and *WNT4*, resulting in PTA formation (Park et al., 2007; Ramalingam et al., 2018). One signaling pathway that amplifies β-catenin activity is Fat4/Hippo signaling from stromal cells. Fat4, by phosphorylation of YAP/TAZ, stimulates transcription of differentiation-related genes (Das et al., 2013). Fat4 binds to Dchs1 in the CM and regulates the polarity of polarized cells. This process is thought to regulate cell-cell communication and cell fate determination (Saburi et al., 2008; Mao et al., 2015). In the following steps of nephrogenesis, WNT4, through a Ca^2+^-dependent pathway, stimulates expression of differentiation genes *FGF8, LHX1, PAX8, Notch, RET, ItgA6a, E-cadherin*, and *ZO1* (Valerius and McMahon, 2008; Tanigawa et al., 2011; Park et al., 2012) in the CM and provokes MET in NPCs. WNT11 is expressed in UB tips through non-canonical pathways and regulates the polarity and behavior of NPCs, which ultimately determines the proper nephrogenic program (O’Brien et al., 2018). During nephrogenesis, some molecules act to downregulate WNT signaling to arrive at an appropriate number of nephrons in a definitive kidney. Dickkopf-1 (DKK1) is an inhibitor of the WNT co-receptor LRP5/6 and downstream of LHX1. During nephrogenesis, DKK1 is expressed by PTA cells and their derivatives (Potter et al., 2007). Stromal cells generate SFRP1, a secreted WNT antagonist that blocks canonical WNT signaling, and restricts NPC differentiation (Levinson et al., 2005).

### Growth Factor Signaling Cross-Talk

Studies of the role of BMPs in kidney organogenesis indicate that BMP2/4 signaling has a critical role in size determination and patterning of the nephrogenic field (Oxburgh et al., 2014). Likewise, BMP7 promotes survival and self-renewal of NPCs. BMP7 is exclusively expressed in the NPCs and UB tips (Blank et al., 2009; Jeanpierre et al., 2012). UB-derived WNT9b induces NPCs expression of BMP7 (Park et al., 2012). The BMP7/SMAD1/5 signaling pathway promotes susceptibility of NPCs to the differentiation signal of WNT9b/β-catenin (Brown et al., 2013; Muthukrishnan et al., 2015). Furthermore, SMAD1 can bind to β-catenin to form a transcriptional activating complex in the promoter region of MYC, and thereby exhibit synergistic effects with the WNT/β-catenin pathway (Hu and Rosenblum, 2005). Decorin, an ECM protein produced by stromal progenitor cells accumulates in the ECM microenvironment that surrounds the NPCs. Decorin antagonizes BMP7/SMAD signaling in NPCs and represses the differentiation signal of the canonical WNT9b/β-catenin pathway. Therefore, ECM components mediate differentiation of NPCs to epithelial structures (Fetting et al., 2014; Ohmori et al., 2015).

BMP7 activates the proliferative signal mediated by the TAK1-JNK-JUN cascade in self-renewing CITED1^+^/SIX2^+^ NPCs. JUN is a DNA-binding partner in the dimeric AP-1 transcription factor. Besides, the activator protein 1 (AP1) also includes another component named FOS. FOS activation is regulated by FGF9 (Muthukrishnan et al., 2015). FGFs is produced in UB cells. Likewise, FGF9 and FGF20 are exclusively expressed in the CM (Jeanpierre et al., 2012). FGF9 expression in these cells is activated by UB-secreted WNT9b. AP1 acts as a point of collaboration between BMP7 and FGF9 signaling pathways. AP-1 activates transcription of the target genes *MYC, BCL-2*, and *p53*, and thereby regulates the cell cycle and proliferation of NPCs (Couillard and Trudel, 2009; Muthukrishnan et al., 2015; Saifudeen et al., 2012). When FGF2 and FGF9 bind to their receptors on the NPCs, the RAS-MAPK and PI3K/AKT signaling cascades are activated, which promotes survival and proliferation of CITED1^+^/SIX2^+^ NPCs (Brown et al., 2011; Lindström et al., 2015). The use of BMP7 and FGFs in directed differentiation of PSCs promotes both proliferation and differentiation of NPCs *in vitro* (Morizane et al., 2015; Taguchi and Nishinakamura, 2017). Recently, researchers have demonstrated that the activin/GDF11/TGFb-SMAD2/3 signaling cascade showed superior effects to BMP7 in maintenance of hiPSC-derived NPCs (Yamamoto et al., 2019).

### Other Signaling Pathways in Nephron Progenitor Cell (NPC) Fate Decision

Notch signaling plays two distinct roles in nephrogenesis. (1) Notch2 downregulates PAX2, SIX2, and GDNF expressions, and thereby primes NPCs for differentiation (Yuri et al., 2015; Chung et al., 2016). (2) Notch is required for accurate segmentation of the nephrons by transcriptional activation of the *LHX1* and *HNF1B* genes (Chung et al., 2017).

The Hedgehog (Hh)/*GLI3R* signaling pathway controls the development of capsular stromal cells by increasing expression of the stromal genes *FOXD1, RALDH2*, and *PBX1*. Furthermore, HH-GLI3R signaling regulates the expression of TGFβ2 and its targets in FOXD1^+^ stromal cells. TGFβ2 that is secreted from the stroma mediates crosstalk between stromal and nephrogenic compartments. In NPCs, TGFβ2 is required for the expression of MET-related genes such as LHX1(Rowan et al., 2018).

## Developmental Events During Ureteric Bud (UB) and Collecting Duct Morphogenesis

Many studies have revealed the critical roles for growth factors secreted from the MM in UB branching morphogenesis (Figure 3). GDNF/RET/glial cell line derived neurotrophic factor family receptor α1 (GFRα1) signaling plays an important role in early developmental events during UB and collecting duct morphogenesis. GDNF secreted from NPCs stimulates cell proliferation and survival in these cells (Pepicelli et al., 1997; Shakya et al., 2005). The positive feedback loop between WNT11 and GDNF/Ret provides for dense packing of the UB branches (Iber et al., 2019). VEGF-A is involved in mitotic proliferation and migration of endothelial and epithelial cells, and may serve to coordinate the formation of blood vessels and kidney tubules during kidney development (Marlier et al., 2008). In the early stages of kidney organogenesis, VEGF-A is produced by NPCs. This molecule influences two adjacent cell populations: Flk1-expressing angioblasts and UB cells (Gao, 2005). In the UB cells, VEGF-A promotes the formation of a Neuropilin 1 and KDR complex, thereby promoting branching morphogenesis in a PKC, ERK1/2, and PI3-K dependent manner (Karihaloo et al., 2005). Furthermore, VEGF-A induces RET activation; therefore, VEGF-A and GDNF have increasing effects on UB cell proliferation and branching morphogenesis (Tufro et al., 2007). These cells send an unknown signal to NPCs to maintain PAX2 and GDNF expressions and, in turn, stimulate branching of the UB (Gao, 2005).

FGF7 and FGF10 are expressed in cortical stromal cells and bind to FGFR2 (IIIb) on UB cells, thereby stimulating UB cell proliferation (Qiao et al., 1999; Ohuchi et al., 2000; Walker et al., 2017). Spatial expression of Ret in the UB branch tips is under the control of stromal-specific transcription factors FOXD1, Rara, Rarb2, and Pod1 (Piscione and Rosenblum, 2002). RA signaling in FOXD1^+^ stromal cells induces the expression and secretion of the extracellular matrix 1 (Ecm1), which restricts expression of Ret to the UB tips (Paroly et al., 2013). Also, SFRP1 from stromal cells may directly down-regulate WNT11 and restrict branching morphogenesis (Yoshino et al., 2002). Sprouty 1/2 and SLIT2/ROBO2 signals restrict UB formation to the posterior nephric duct (Grieshammer et al., 2004; Licht et al., 2005; Wilhelm et al., 2015). Several factors such as BMP4 and TGFβ inhibit UB elongation (Cain et al., 2005; Lopez-Rios et al., 2007; Sakurai and Nigam, 2017) and can generate a proper definitive renal collecting system structure and position.

## Endothelial Migration and Patterning During Renal Vascular Development

**FIGURE 4:**
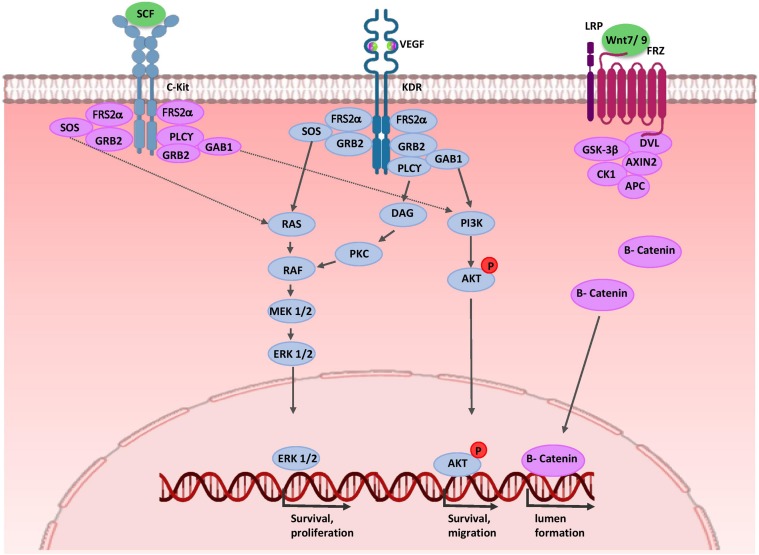
Molecular control of renal endothelial migration and patterning. VEGF is secreted by UB and NPC cells. Binding of VEGF to its tyrosine kinase receptor (KDR) activates three major signaling pathways: RAS/MAPK, DAG/PKC/MAPK, and PI3-K/AKT. Thus, it stimulates mitotic proliferation, survival, and migration of endothelial cells and promotes vascular network formation. UB cells produce SCF and induce survival, migration, and tube formation of endothelial cells. Expressions of WNT7b and WNT9b in the medullary ureteric epithelium regulate capillary lumen formation through modulation of VE-cadherin localization. AKT, protein kinase B; APC, adenomatous polyposis coli; c-Kit, tyrosine-protein kinase KIT (CD117); CK1, casein kinase 1; DAG, diacylglycerol; EC, endothelial cells; ERK, extracellular signal-regulated kinase; FRS2α, fibroblast growth factor receptor substrate 2; Frz, Frizzled; GAB1, Grb2-associated binder 1; GSK-3β, glycogen synthase kinase 3β; GRB2, growth factor receptor-bound protein 2; KDR, kinase insert domain protein receptor; MAPK, mitogen activated protein kinase; MEK, mitogen activated protein kinase; P, phosphate group; PI3K, phosphatidylinositol 3-kinase; PKC, protein kinase C; PLC-γ, phospholipase C-γ; RAS/RAF, Rat sarcoma/rapidly accelerated fibrosarcoma; SCF, stem cell factor; SOS, Son of Sevenless; VEGF, vascular endothelial growth factor; WNT, wingless-type mouse mammary tumor virus integration site.

As mentioned before, NPCs and UB cells produce VEGF-A (Gao, 2005; Marlier et al., 2008). VEGF-A binds to VEGFR-2 (KDR, Flk-1) on the EPC surface and the signal transduction events activate endothelial progenitor mitotic proliferation and migration (Abrahamson et al., 1998). At later stages, presumptive podocytes in the S-shaped bodies express VEGF-A and recruit EC into the developing glomerulus (Mundel et al., 2003; Eremina, 2006). Activation of ECs may involve a signaling pathway independent of VEGF. UB cells secrete SCF, a c-Kit ligand, and thereby promote survival, migration, and tube formation of ECs (Figure 4) (Schmidt-ott et al., 1993; Homan et al., 2019). Expressions of WNT7b and WNT9b in the medullary ureteric epithelium regulate capillary lumen formation through modulation of VE-cadherin localization (Roker et al., 2017).

Stromal cells have critical roles in the normal hierarchical pattern of the renal vasculature. It is thought to that adjacent stromal cells secret SFRP1, and thereby induce proliferation, migration and tubulogenesis of ECs (Dufourcq et al., 2002; Yoshino et al., 2002). Recombination signal binding protein for immunoglobulin kappa J region (RBP-J)-mediated Notch signaling regulates vascular patterning by controlling stromal progenitor differentiation into the vascular mural cell layer of the renal arteries and mesangial cells (Lin and Gomez, 2014). At later stages, EC-derived PDGF-β binds to platelet-derived growth factor receptor beta (PDGFRβ) on stromal cells; thereby, ECs are recruited into the glomerulus and generate capillary loops. On the other hand, in stromal cells, PBX1 temporally and spatially restrict PDGFRβ expression patterns to cortical domains of the kidney, leading to renal vascular stabilization (Hurtado et al., 2015; Daniel and Cleaver, 2019). Finally, perivascular macrophages in the nephrogenic zone interact with newly forming renal vessels and promote vascular anastomoses. Thus, they play a critical role in proper vessel network formation (Munro et al., 2019).

## Kidney Organoids: Translating Developmental Knowledge Into the Dish

Many recent efforts have aimed to generate *in vitro* 3D models of both functional tissues and organs to study human developmental and physiological processes, drug screening, disease modeling, and regenerative medicine applications. A defined culture system drives forward the differentiation of human PSCs into kidney organoids by recapitulating the developmental signaling events. Human metanephric kidney formation is initiated during the fifth week of gestation and this corresponds to embryonic day 10.5 (E10.5) for mouse kidney formation (Reidy and Rosenblum, 2009; Costantini and Kopan, 2010). Human PSC-derived kidney organoids, like the developing kidney, should be composed of NPCs, UBPCs, and stromal and EPCs. Recently, scientists have developed protocols that mimic kidney developmental paths *in vivo*. A cocktail of small molecules and growth factors (CHIR99021, Noggin, Activin A, FGF9, and BMP7) are essential for *in vitro* renal lineage differentiation (Morizane et al., 2015; Ahmadi et al., 2019b; Mansoori-moghadam et al., 2019). The stepwise processes of directed differentiation includes intermediate cell populations: mesendoderm, PIM, RPCs, PTAs, RVs, and the mature kidney. Next, we describe protocols and methods used for the generation of *in vitro* 3D kidney organoids and how developmental knowledge can improve their complexity.

### Kidney Organoid Differentiation Protocols and Methods

Takasato and colleagues have reported a protocol that generates human iPSC-derived kidney organoids that consist of both NPCs and UBPCs-derived populations, as well as the CD31^+^/KDR1^+^/SOX17^+^ endothelial network, and cortical (FOXD1^+^/MEIS1^+^) and medullary (FOXD1^+^/MEIS1^+^) stromal cells. RNA sequencing analysis indicated that Takasato organoids were very similar to the first trimester kidney (Takasato et al., 2015). Several studies utilized the Takasato protocol to conduct developmental studies (Bantounas et al., 2018), disease modeling (Forbes et al., 2018), *in vivo* transplantation (Koning et al., 2018), and a scale-up of organoid generation (Kumar et al., 2019). Another study was conducted by Bonventre’s laboratory. Their method, the Morizane differentiation protocol, enabled 75–92% induction efficiency of NPCs with a shorter differentiation period from hPSCs. However, the nephron derived organoids lacked UB lineages (Morizane and Bonventre, 2017). Wu and colleagues compared the Takasato and Morizane differentiation protocols by using single-cell transcriptomics of hPSCs-derived kidney organoid cells. Their data demonstrated that both protocols generated organoids with at least 12 individual kidney cell types, but with different proportions. Both protocols produced non-renal cells including neurons, muscles, and melanocytes. The Morizane protocol generated only 11% non-renal cells whereas the Takasato organoids contained about 21% of these cells. Although hPSCs-derived kidney organoids expressed some markers of terminal differentiation, they were relatively immature. The Morizane protocol had fewer proliferative cells, and their organoids had more podocyte cells and more differentiated loop of Henle, whereas the Takasato protocol generated more tubular epithelial cell types (Wu et al., 2018).

In another study, hPSCs cultured between two layers of dilute Matrigel (0.2 mg ml), and cavitated epiblast spheroids were produced. To induce the differentiation of the spheroids toward kidney organoids, the researchers used GSK-3β inhibitor CHIR99021 (12 μM) for 1.5 days, and then incubated the spheroids in B27-supplemented media for up to 16 days. The spheroids underwent a sequential epithelial-mesenchymal transition (EMT) and mesenchymal-epithelial transition (MET) process and acquired 3D kidney structures that contained some segments of nephron such as PODXL^+^/WT1^+^/SYNPO^+^ podocytes, lotus tetragonolobus lectin (LTL)^+^ proximal tubules, and immature ECs (Siedlecki et al., 2015). These organoids resembled an immature kidney reminiscent of the late first trimester to mid-second trimester human kidney. Podocyte cells are similar to developing capillary loop stage podocytes *in vivo* (Brooks et al., 2017; Harder et al., 2019) and their nephron-like organoids contain non-renal cells, including ectoderm and lateral plate mesoderm derivatives (Morizane and Bonventre, 2017). In another study, the same protocol was used to generate hPSC-derived organoid plates in microwell formats (high-throughput screening platform) to enhance the differentiation efficiency of the kidney organoids. Single-cell RNA sequencing analysis revealed six major clusters of cell type subpopulations that included proximal tubules, podocytes, early tubules that expressed markers of both proximal and distal tubules and collecting ducts, early podocytes that had characteristics of both CLDN1^+^/PAX8^+^ parietal epithelial cells and podocytes, as well as, stromal and ECs (Czerniecki et al., 2018).

In a recent study, Garreta and colleagues improved the speed and efficiency of maturation by increasing the duration of the 3D culture. They utilized soft hydrogels during the monolayer culture for 4 days to derive IM committed cells that contained both AIM and PIM cell populations. Afterward, the cells were aggregated in a 96-well V-bottom plate to form the 3D structures. Aggregates were cultured in the presence of the induction factors for 16 days to generate the kidney organoids. Their hPSCs-derived kidney organoids contained various kidney cell types, including cell populations with characteristics of proximal tubules, loops of Henle, distal tubules, and glomeruli. The human kidney organoids were transplanted into chick chorioallantoic membrane (CAM) and incubated *in ovo* for 5 days. hPSCs-derived organoid transplants showed more *in vivo*-like characteristics with higher functional differentiation compared to *in vitro* organoids, and transcriptionally, they more closely resembled second trimester human fetal kidneys (Garreta et al., 2019). Hiratsuka et al. (2019) established a new approach that used synthetic mRNAs to generate induced nephron-like organoids (iNephLOs) with some segments of the nephron that were comprised of proximal tubules, distal tubules, and podocytes. They used two sets of synthetic mRNAs encoding transcription factors for 4 days to derive PAX8^+^/LHX1^+^ pretubular aggregate cells, after which the cells were aggregated in 96-well U-bottom plate for up to 14 days to form 3D structures that transcriptionally resembled kidney organoids generated by using growth factor induction (Hiratsuka et al., 2019).

There is a large variation between independent differentiation experiments. The variability may arise from the technical strategies underlying kidney organoids formation such as inter-reagent and inter-batch variability, variation between hPSC lines, and even skill of the experimenter. Moreover, the generation of hPSC-derived kidney organoids faces many remaining challenges including immature renal cell types, nascent vascular network, and lack of connection between nephron segments and collecting duct system in organoids (Miyoshi et al., 2019). Therefore, developing more *in vivo* mimicking structures by biological approaches for reproducible and robust generation of kidney organoid is urgently needed.

### Biological Approaches to Improve Kidney Organoid Complexity

Cell-to-cell interactions and signals from the complex microenvironment during embryonic kidney development affect cell behaviors such as proliferation, migration and differentiation of renal progenitors. Organoids can be generated following the same developmental events that occur in the embryo; thus, consideration for cellular communications and microenvironmental cues in organoids can improve their complexity.

#### Cell-to-Cell Interactions

Like early organogenesis event, a tightly coordinated crosstalk between NPCs, UBPCs, ECs, and stromal cells during development leads to a higher-order and vascularized kidney organoid (Takasato et al., 2016). Studies have shown that increasing the duration of the 3D culture by enhancing cell–cell and cell–matrix interactions would generate kidney organoids with higher maturity (Garreta et al., 2019). Most studies of kidney organoids have relied on the self-organizing capacity of aggregates derived from uni-lineage progenitors such as hPSCs (Morizane et al., 2015; Takasato et al., 2015; Garreta et al., 2019). However, some studies have developed heterotypic cellular aggregates. Taguchi and Nishinakamura established a protocol for differential induction of mouse NPCs and UBPCs separately, and reaggregated them with the PDGFRa^+^ stromal progenitors isolated from E11.5 mouse embryonic kidneys. This reassembled kidney organoid had more differentiated nephron structures with overall components and contiguous collecting duct architecture (Taguchi and Nishinakamura, 2017). Many studies, including researches in our laboratory, indicated that when hPSC-derived progenitor cells were combined with mesenchymal stem cells (MSCs) and ECs, the mixture self-organized into 3D structures such as kidney (Takebe et al., 2015), pancreatic (Takebe et al., 2015; Takahashi et al., 2018), cardiac (Varzideh et al., 2018), and liver (Takebe et al., 2014, 2015, 2017) organoids. Data showed that stromal cells produced various cytokines and growth factors that modulated the proliferation and maturation of other cells in the organoids (Takahashi et al., 2018). Myosin IIA expressed by MSCs directed forceful movements of cells and triggered the initiation of self-condensation (Takebe et al., 2015).

Research has shown that ECs are important not only for delivery of oxygen and micronutrients, but also for the paracrine signals that are critical for proper RPCs differentiation and promotion of organoid maturation (Garreta et al., 2019). EC generation in both the Takasato and the Morizane differentiation protocols was very low (0.3% or less of total cells) (Wu et al., 2018). A recent study developed a highly-efficient protocol that used a three-step CHIR treatment to generate a subset of SIX1^+^/KDR^+^/PECAM1^+^ NPCs that contributed to new vessel formation in 3D hPSC-derived kidney organoids. VEGF-A secretion by differentiating podocytes within the organoid supported maturation of the newly formed ECs (Low et al., 2019). Some studies demonstrated that VEGF supplementation during the differentiation process resulted in a significant increase of ECs and a population of stromal cells that expressed the VEGF receptor, FLT1, and maturation and maintenance of organoid vasculature. However, many of these ECs have immature characteristics. These ECs fail to invade the developing podocytes (Czerniecki et al., 2018; Koning et al., 2018). Moreover, organoid transplantation into a highly vascularized site such as the sub-renal capsule and CAM facilitates both vascularization and maturation of organoids (Koning et al., 2018; Varzideh et al., 2018; Garreta et al., 2019; Low et al., 2019).

#### Microenvironmental Cues

Microenvironmental cues include biochemical and biophysical (oxygen tension, ECM stiffness, and fluid flow) signals during organogenesis regulate cell behavior of different stem cell populations (Vining and Mooney, 2017; Silva et al., 2019). The ECM of the kidney is a complex architectural network that contains collagens, elastin, and several proteoglycans and glycoproteins, which together form basal membranes and the interstitial space. In addition to its biochemical cues, these dynamic structures provide mechanical support and mediate the cell signaling pathways, which are essential for proper kidney development and function (Bülow and Boor, 2019).

Researchers have demonstrated that cells sense the ECM stiffness by mechanoreceptors such as integrins. Thus, the ECM plays a key role in the cell fate decision (Akkerman and Defize, 2017). ECM stiffness regulates differentiation into each germ layer (Zoldan et al., 2011). Researchers have sought to determine if the substrate matrix stiffness may affect self-organization and maturation of the organoids. Takebe and colleagues used a co-culture system by combining hPSC-derived tissue-specific progenitors with MSCs and ECs. This mixture was transferred onto Matrigel with varying degrees of stiffness. Data showed that self-condensation was promoted by soft environmental conditions (*E* ∼ 10 ∼ 20 kPa) in their 3D culture system (Takebe et al., 2015). Garreta and colleagues fabricated polyacrylamide hydrogels with varying mechanical properties (1–60 kPa). They investigated whether substrates with mechanical properties similar to native tissues such as CAM could improve organoid maturation. RNA-Seq analysis revealed that the soft substrate (*E*∼1 kPa) improved the expressions of mesodermal lineage genes *T, PAX2, SALL1, LHX1*, and *Hoxd11*. These organoids had more mature features when compared with rigid conditions (Garreta et al., 2019).

Fluid flow is a mechanical force that plays a key role in the developmental process, including vascularization and differentiation (Ghaffari et al., 2015; Vining and Mooney, 2017). Homan et al. (2019) have investigated the effect of fluid shear stress (FSS) in vascularization and maturation of hPSCs-derived kidney organoids. hPSCs were differentiated into pretubular aggregate cells as previously reported (Morizane et al., 2015). They placed these aggregates onto a gelatin-fibrin (gelbrin) ECM layer within a 3D-printed millifluidic chip that was perfused under varying flow rates for 10 days. Their data showed that gelbrin increased expression of endothelial markers PECAM1 and MCAM. Under high FSS at differentiation day 21, they observed significantly enhanced expansion and differentiation of the KDR^+^ and PECAM1^+^ ECs with formation of perfusable vascular anastomosis between the organoids. As the organoid vasculature evolved, PDGFRβ^+^ pericyte-like cells greatly increased in numbers and were recruited to the vascular network. Accordingly, endothelial-epithelial crosstalk increased the maturation of tubular and glomerular cells within the kidney organoids in comparison to static conditions (Homan et al., 2019).

## Conclusion and Perspectives

Major advances have been made in the understanding of various cellular components and intercellular signaling pathways involved in kidney development. The creation of kidney organoids from hPSCs by assessing the cells at each step of nephrogenesis has also expanded our knowledge of kidney development. Organoids can be generated following the same developmental events that occur in the embryo. Thus, consideration for cellular communications and microenvironmental cues in organoids can improve their complexity. Despite some significant improvements, there are difficult challenges that remain before this technology can be used in modern regenerative medicine. Until now, kidney organoids have been in a relatively immature state comparable to fetal nephrons. Moreover, kidney organoid vasculature is not fully mature, and their nascent ECs fail to invade the glomerular primordial. In the future, kidney organoids combined with recent biotechnological progresses such as microfluidic kidney-on-a-chip, co-culture systems, and 3D bioprinting technology have the potential to revolutionize developmental studies, drug screening, and personalized medicine.

## Author Contributions

NK contributed to the preparation of this manuscript for writing and conducting the literature review. RM and NA drafted the manuscript and revised it critically for all content.

## Conflict of Interest

The authors declare that the research was conducted in the absence of any commercial or financial relationships that could be construed as a potential conflict of interest.

## Abbreviations

3D: three-dimensional
AIM: anterior intermediate mesoderm
AP1: activator protein 1
BMP: bone morphogenetic protein
CITED1: Cbp/p300-interacting transactivator 1
CM: cap mesenchyme
DKK1: Dickkopf-1
ECM: extracellular matrix
Ecm1: extracellular matrix 1
EC: endothelial cells
EMX: empty spiracles homolog
EPC: endothelial progenitor cells
ERK: extracellular signal-regulated kinase
Eya1: eyes absent 1
FGF: fibroblast growth factor
FOXD1: forkhead/winged helix transcription factor
FSS: fluid shear stress
GATA: *trans*-acting T-cell -specific transcription factor
Gas1: growth arrest-specific 1
GDNF: glial cell-derived neurotrophic factor
GFR α 1: glial cell line derived neurotrophic factor family receptor α 1
Hox: homeobox
hPSCs: human pluripotent stem cells
IM: intermediate mesoderm
Kdr/VEGFR2/Flk1: kinase insert domain protein receptor
LHX1: LIM-class homeodomain 1
LTL: lotus tetragonolobus lectin
MCAM: melanoma cell adhesion molecule (CD146)
MET: mesenchymal-epithelial-transition
MM: metanephric mesenchyme
MSCs: mesenchymal stem cells
NPCs: nephron progenitor cells
Odd1 or Osr1: odd skipped related 1
PAX: paired box protein
PBX1: Pre-B-cell leukemia transcription factor 1
PDGFRβ: Platelet-derived growth factor receptor beta
PECAM-1/CD31: Platelet/endothelial cell adhesion molecule-1
PIM: posterior intermediate mesoderm
PI3K: Phosphoinositide 3-kinase
PTA: pre-tubular aggregate
RA: retinoic acid
RAR: retinoic acid receptor
RBP-J: recombination signal binding protein for immunoglobulin kappa J region
RV: renal vesicle
SALL1: spalt like transcription factor 1
SCF: stem cell factor
SFRP: secreted frizzled-related proteins
SIX2: sine oculis-related homeobox 2
TGFβ: transforming growth factor beta
UB: ureteric bud
UBPCs: ureteric bud progenitor cells
VEGF: vascular endothelial growth factor
VEGFR: vascular endothelial growth factor receptor
WT1: Wilm’s tumor
WNT: wingless-type mouse mammary tumor virus integration site.
